# The CSF p-tau/β-amyloid 42 ratio correlates with brain structure and fibrillary β-amyloid deposition in cognitively unimpaired individuals at the earliest stages of pre-clinical Alzheimer’s disease

**DOI:** 10.1093/braincomms/fcae451

**Published:** 2024-12-13

**Authors:** Raffaele Cacciaglia, Mahnaz Shekari, Gemma Salvadó, Marta Milà-Alomà, Carles Falcon, Gonzalo Sánchez-Benavides, Carolina Minguillón, Karine Fauria, Oriol Grau-Rivera, José Luis Molinuevo, Kaj Blennow, Henrik Zetterberg, Frances-Catherine Quevenco, Marc Suárez-Calvet, Juan Domingo Gispert, the ALFA Study, Ricardo A Aguilar, Annabella B Gorriti, Anna B Serrat, Raffaele Cacciaglia, Lidia C Gispert, Alba C Martinez, Marta D Milan, Carmen D Gomez, Ruth D Iglesias, Marie E F Karine, Sherezade F Julian, Patricia G Serra, Juan D Gispert, Armand G Escalante, Oriol G Rivera, Laura H Penas, Gema H Rodriguez, Jordi H Ninou, Laura I Gamez, Iva Knezevic, Paula M Alvarez, Tania M Diaz, Carolina M Gil, Eva Palacios, Maria Pascual, Albina P Ballester, Sandra P Mendez, Irina A Radoi, Blanca R Fernandez, Laura R Freixedes, Aleix S Vila, Gonzalo A Sanchez Benavides, Mahnaz S Mahnaz, Lluis S Harster, Anna S Prat, Laura S Stankeviciute, Marc S Calvet, Marc V Jaramillo, Natalia V Tejedor, Annabella Beteta, Alba Cañas, Carme Deulofeu, Irene Cumplido, Ruth Dominguez, Maria Emilio, Sherezade Fuentes, Laura Hernandez, Gema Huesa, Jordi Huguet, Paula Marne, Tania Menchón, Albina Polo, Sandra Pradas, Anna Soteras, Marc Vilanova

**Affiliations:** Barcelonaβeta Brain Research Center (BBRC), Pasqual Maragall Foundation, Barcelona 08005, Spain; Hospital del Mar Medical Research Institute (IMIM), Barcelona 08005, Spain; Centro de Investigación Biomédica en Red de Fragilidad y Envejecimiento Saludable (CIBERFES), Madrid 28089, Spain; Barcelonaβeta Brain Research Center (BBRC), Pasqual Maragall Foundation, Barcelona 08005, Spain; Hospital del Mar Medical Research Institute (IMIM), Barcelona 08005, Spain; Centro de Investigación Biomédica en Red de Fragilidad y Envejecimiento Saludable (CIBERFES), Madrid 28089, Spain; Barcelonaβeta Brain Research Center (BBRC), Pasqual Maragall Foundation, Barcelona 08005, Spain; Hospital del Mar Medical Research Institute (IMIM), Barcelona 08005, Spain; Centro de Investigación Biomédica en Red de Fragilidad y Envejecimiento Saludable (CIBERFES), Madrid 28089, Spain; Department of Clinical Sciences, Clinical Memory Research Unit, Lund University, Box 117, SE-221 00 Lund, Sweden; Barcelonaβeta Brain Research Center (BBRC), Pasqual Maragall Foundation, Barcelona 08005, Spain; Hospital del Mar Medical Research Institute (IMIM), Barcelona 08005, Spain; Centro de Investigación Biomédica en Red de Fragilidad y Envejecimiento Saludable (CIBERFES), Madrid 28089, Spain; Barcelonaβeta Brain Research Center (BBRC), Pasqual Maragall Foundation, Barcelona 08005, Spain; Hospital del Mar Medical Research Institute (IMIM), Barcelona 08005, Spain; Centro de Investigación Biomédica en Red de Bioingeniería, Biomateriales y Nanomedicina (CIBERBBN), Madrid 28089, Spain; Barcelonaβeta Brain Research Center (BBRC), Pasqual Maragall Foundation, Barcelona 08005, Spain; Hospital del Mar Medical Research Institute (IMIM), Barcelona 08005, Spain; Centro de Investigación Biomédica en Red de Fragilidad y Envejecimiento Saludable (CIBERFES), Madrid 28089, Spain; Barcelonaβeta Brain Research Center (BBRC), Pasqual Maragall Foundation, Barcelona 08005, Spain; Hospital del Mar Medical Research Institute (IMIM), Barcelona 08005, Spain; Centro de Investigación Biomédica en Red de Fragilidad y Envejecimiento Saludable (CIBERFES), Madrid 28089, Spain; Barcelonaβeta Brain Research Center (BBRC), Pasqual Maragall Foundation, Barcelona 08005, Spain; Hospital del Mar Medical Research Institute (IMIM), Barcelona 08005, Spain; Centro de Investigación Biomédica en Red de Fragilidad y Envejecimiento Saludable (CIBERFES), Madrid 28089, Spain; Barcelonaβeta Brain Research Center (BBRC), Pasqual Maragall Foundation, Barcelona 08005, Spain; Hospital del Mar Medical Research Institute (IMIM), Barcelona 08005, Spain; Centro de Investigación Biomédica en Red de Fragilidad y Envejecimiento Saludable (CIBERFES), Madrid 28089, Spain; Servei de Neurologia, Hospital del Mar, 08005 Barcelona, Spain; Lundbeck A/S, 2500 Copenhagen, Denmark; Department of Psychiatry and Neurochemistry, Institute of Neuroscience and Physiology, The Sahlgrenska Academy at the University of Gothenburg, Mölndal 43180, Sweden; Clinical Neurochemistry Laboratory, Sahlgrenska University Hospital, Mölndal 43180, Sweden; Department of Psychiatry and Neurochemistry, Institute of Neuroscience and Physiology, The Sahlgrenska Academy at the University of Gothenburg, Mölndal 43180, Sweden; Clinical Neurochemistry Laboratory, Sahlgrenska University Hospital, Mölndal 43180, Sweden; UK Dementia Research Institute at University College London, London WC1E 6BT, UK; Department of Neurodegenerative Disease, UCL Institute of Neurology, London WC1N 3BG, UK; Hong Kong Center for Neurodegenerative Diseases, Hong Kong, China; UW Department of Medicine, School of Medicine and Public Health, Madison, WI 53705-2281, USA; Roche Diagnostics International Ltd, 6343 Rotkreuz, Switzerland; Barcelonaβeta Brain Research Center (BBRC), Pasqual Maragall Foundation, Barcelona 08005, Spain; Hospital del Mar Medical Research Institute (IMIM), Barcelona 08005, Spain; Centro de Investigación Biomédica en Red de Fragilidad y Envejecimiento Saludable (CIBERFES), Madrid 28089, Spain; Servei de Neurologia, Hospital del Mar, 08005 Barcelona, Spain; Barcelonaβeta Brain Research Center (BBRC), Pasqual Maragall Foundation, Barcelona 08005, Spain; Hospital del Mar Medical Research Institute (IMIM), Barcelona 08005, Spain; Centro de Investigación Biomédica en Red de Bioingeniería, Biomateriales y Nanomedicina (CIBERBBN), Madrid 28089, Spain; Departament de Medicina i Ciències de la Vida, Universitat Pompeu Fabra, Barcelona 08002, Spain

**Keywords:** Alzheimer’s disease, CSF biomarkers, p-tau/Aβ42 ratio, brain structure, amyloid deposition

## Abstract

CSF concentrations of β-amyloid 42 (Aβ42) and phosphorylated tau (p-tau) are well-established biomarkers of Alzheimer’s disease and have been studied in relation to several neuropathological features both in patients and in cognitively unimpaired individuals. The CSF p-tau/Aβ42 ratio, a biomarker combining information from both pathophysiological processes, has emerged as a promising tool for monitoring disease progression, even at pre-clinical stages. Here, we studied the association between the CSF p-tau/Aβ42 ratio with downstream markers of pre-clinical Alzheimer’s disease progression including brain structure, glucose metabolism, fibrillary Aβ deposition and cognitive performance in 234 cognitively unimpaired individuals, who underwent cognitive testing, a lumbar puncture, MRI, 18F-fluorodeoxyglucose and 18F-flutemetamol PET scanning. We evaluated both main effects and interactions with Alzheimer’s disease risk factors, such as older age, female sex and the apoliporoptein E (*APOE*)-ɛ4 allele, in *a priori* defined regions of interest and further examined the associations on the whole-brain using voxel-wise regressions. In addition, as the association between CSF Alzheimer’s disease biomarkers and brain structure and function may be non-linear, we tested the interaction between the CSF p-tau/Aβ42 ratio and stages of pre-clinical Alzheimer’s disease defined using the amyloid (A) and tau (T) classification. We found significantly positive associations between CSF p-tau/Aβ42 and both cortical Aβ deposition and regional grey matter volume while no effect was observed for brain metabolism. A significant interaction with age indicated that, for the same level of CSF p-tau/Aβ42, older individuals displayed both increased Aβ deposition and lower grey matter volume, in widespread cortical areas. In addition, we found that women compared with men had a greater Aβ fibrillary accumulation in midline cortical areas and inferior temporal regions, for the same level of the CSF biomarker. The impact of CSF p-tau/Aβ42 on grey matter volume was modulated by AT stages, with A+T+ individuals displaying significantly less positive associations in areas of early atrophy in the Alzheimer’s continuum. Finally, we found that sex and *APOE*-ɛ4 modulated the association between the CSF biomarker and episodic memory as well as abstract reasoning, respectively. Our data indicate that the CSF p-tau/Aβ42 ratio is strongly associated with multiple downstream neuropathological events in cognitively unimpaired individuals and may thus serve as a potent biomarker to investigate the earliest changes in pre-clinical Alzheimer’s disease. Given that its impact on both Aβ deposition and grey matter volume is modulated by specific risk factors, our results highlight the need to take into account such predisposing variables in both clinical practice and prevention trials.

## Teaser

Cacciaglia *et al*. report that the CSF phosphorylated tau/amyloid-β (Aβ) 42 ratio is associated with brain structure and fibrillary Aβ deposition in cognitively unimpaired individuals. These findings suggest that the biomarker is useful for monitoring early stages of Alzheimer’s disease, with effects modulated by risk factors such as age, sex, and *APOE*-ɛ4 status.

## Graphical Abstract

**Graphical Abstract:**
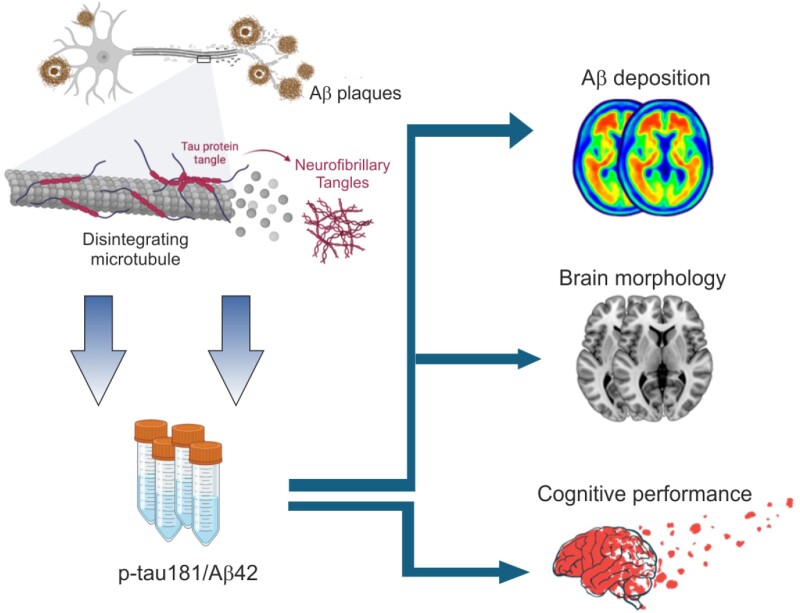

## Introduction

Alzheimer’s disease, the most common form of dementia, is characterized by an insidious onset, where multiple histopathological changes precede any detectable clinical symptoms by decades.^1^ A decrease in CSF concentrations of amyloid-β (Aβ) 42 is considered the earliest event in the Alzheimer’s disease pathophysiological cascade, which is followed by CSF phosphorylated tau (p-tau) increase and cortical atrophy along with cognitive decline.^2^ Understanding the contribution of Aβ and tau pathology to downstream neuropathological events across the Alzheimer’s *continuum* is a key for the identification of disease mechanisms and is critical for identifying early therapeutic approaches. In cognitively unimpaired (CU) individuals, Aβ pathology has been associated with both increased^3-6^ and decreased^7-10^ grey matter volume (GMV) or cortical thickness. The nature of this association may depend on genetic risk factors,^11,12^ the cooccurrence of tau pathology^13,14^ and the disease stage where this association is being tested.^15,16^ By contrast, abnormal p-tau has been consistently associated with decreased GMV across different stages in the Alzheimer’s *continuum.*^17-19^

The CSF p-tau/Aβ42 ratio combines two different pathological processes into a single measure and provides one of the best diagnostic accuracies, comparable with imaging-based biomarkers such as amyloid PET, as demonstrated by direct head-to-head comparisons in several studies.^20,21^ Head-to-head comparisons among different biomarker types have shown a better performance of the CSF p-tau/Aβ42 ratio over Aβ42 alone in predicting fibrillary Aβ deposition, both in cognitively unimpaired (CU) individuals^22,23^ and patients with mild cognitive impairment.^24,25^ Similarly, the CSF p-tau/Aβ42 ratio displayed higher specificity in predicting conversion from mild cognitive impairment to Alzheimer’s disease compared with other biomarkers such as Aβ42, total tau (t-tau) and phosphorylated tau181 (p-tau181), with specificity values of 90^26^ and 95%.^27^ The superiority of this hybrid biomarker may be due to several reasons. Incorporating p-tau alongside Aβ42 may provide a more accurate reflection of fibrillary Aβ plaque deposition, as p-tau adds sensitivity to the early detection of Aβ pathology.^28,29^ In addition, adjusting p-tau by Aβ42 concentrations may also reduce random variance in tau production and clearance, which may increase its sensitivity. To date, no study has directly assessed the impact of this combined biomarker on downstream neuropathological events in CU individuals. Therefore, in the present study, we assessed the impact of continuous CSF p-tau/Aβ42 concentrations on brain morphology, brain metabolism and fibrillary Aβ deposition, as well as cognitive performance, in a sample of CU individuals at the earliest inception of the Alzheimer’s *continuum*, allowing us to study very early effects associated with disease progression. Furthermore, we sought to determine whether these putative associations were modulated by Alzheimer’s disease risk factors such as older age, female sex and apolipoprotein E (*APOE*)-ɛ4. Finally, to test the previously proposed hypothesis of a non-linear effect of Alzheimer’s pathology on brain structure and function,^15,16^ we tested the interaction between the CSF biomarker and categorical stages of Alzheimer’s pathology in driving GMV and cerebral metabolism.

## Methods

### Study participants

The ALFA study (Clinicaltrials.gov Identifier: NCT01835717) comprises a longitudinal monocentric research platform aimed at the identification of pathophysiological alterations in pre-clinical Alzheimer’s disease. The ALFA cohort is composed of 2743 CU individuals, all having a Clinical Dementia Rating score of 0, with most being first-order descendants of Alzheimer’s disease patients.^30^ Within this research framework, the ALFA+ is a nested study that includes advanced imaging protocols, including MRI and PET acquisitions, along with cognitive testing, measurement of lifestyle factors as well as fluid biomarkers. The ALFA+ study (ALFA-FPM-0311) was approved by the Independent Ethics Committee ‘Parc de Salut Mar’, Barcelona and registered at Clinicaltrials.gov (Identifier: NCT02485730). The present study included the first 234 consecutive participants of the ALFA+ study with available CSF, Aβ PET, 18F-fluorodeoxyglucose (FDG) PET, MRI and cognitive data. The mean follow-up time in ALFA+ is 3.5 years, and we are currently collecting the data of the third follow-up. However, all tests included in the present study are performed with data of the baseline ALFA+ visit, and they are all cross-sectional. None of the subjects had a neurologic or psychiatric diagnosis. The average time between the lumbar puncture (CSF data) and PET scan was 3.71 months (SD = 4.44) and 2.21 months (SD = 3.87) between CSF data and the MRI/neuropsychological evaluation. All participating subjects signed the study’s informed consent form, which was approved by the Independent Ethics Committee ‘Parc de Salut Mar’, Barcelona. The study was conducted according to the Declaration of Helsinki.

### CSF sampling and analysis

CSF was collected by lumbar puncture into a 15 ml sterile polypropylene sterile tube (Sarstedt, Nümbrecht, Germany; cat. no. 62.554.502), following standard procedures.^31^ CSF was aliquoted in volumes of 0.5 ml into sterile polypropylene tubes (0.5 ml Screw Cap Micro Tube Conical Bottom; Sarstedt; cat. no. 72.730.005) and immediately frozen at −80°C. Overall, the time between collection and freezing was <30 min. All determinations were done in aliquots that had never been previously thawed. Aβ42 and Phospho-tau (181P, hereinafter referred as p-tau) concentrations were measured using the Elecsys^®^ electrochemiluminescence immunoassay on the fully automated cobas e601 analyzer (Roche Diagnostics International Ltd.) at the Clinical Neurochemistry Laboratory, University of Gothenburg, Sweden. For all participants, the ratio between CSF Aβ42 and p-tau was calculated to be entered in subsequent statistical models as the independent variable. For descriptive purposes, individuals were classified into subgroups identifying the pathophysiological stages of both CSF Aβ42 (A) and p-tau (T),^32^ using previously validated cut-offs for CSF Aβ42 (A+: <1098 pg/ml) and p-tau (T+: >19 pg/ml).^21^ Out of the original 242 study participants, 8 were classified as A−T+. These individuals were excluded from all subsequent analyses as they are thought to reflect non-Alzheimer’s disease pathological changes,^32^ resulting in a final sample of *n* = 234 study participants.

### Apoliporoptein E genotype

Total DNA was obtained from blood cellular fraction by proteinase K digestion followed by alcohol precipitation. All individuals were genotyped for two single nucleotide polymorphisms, rs429358 and rs7412, to define the *APOE*-ɛ2, ɛ3 and ɛ4 alleles. Of the 234 study participants, 10 were ɛ2/ɛ3, 79 were ɛ3/ɛ3, 7 were ɛ2/ɛ4, 112 were ɛ3/ɛ4 and 26 were ɛ4/ɛ4. In all analyses involving *APOE*-ɛ4 status as a factor, participants were divided into two groups: non-carriers (*i.e.* ɛ2/ɛ3 + ɛ3/ɛ3) and carriers (ɛ2/ɛ4 + ɛ3/ɛ4 + ɛ4/ɛ4).

### MRI data acquisition and pre-processing

A T1-weighted 3D-turbo field echo (TFE) sequence was acquired with a 3T Philips Ingenia CX scanner with the following sequence parameters: voxel size = 0.75 mm isotropic, field of view = 240 × 240 × 180 mm^3^, flip angle = 8°, repetition time = 9.9 ms, echo time = 4.6 ms and time of inversion (TI) = 900 ms. Grey matter (GM) was segmented from images using the new segment function implemented in Statistical Parametrical Mapping software (SPM 12, Wellcome Department of Imaging Neuroscience, London, UK) and located into a common space for subsequent normalization using a 12-affine parameter transformation. Segmented GM images were used to generate a reference template of the sample, which was warped into a standard Montreal Neurological Institute (MNI) space using the high dimensional DARTEL toolbox.^33^ The generated flow fields and normalization parameters were then implemented to normalize the native GM images to the MNI space. In order to preserve the native local amount of GM volume, we applied a modulation step, where each voxel signal’s intensity was multiplied by the Jacobian determinants derived from the normalization procedure.^34^ Quality control of normalization was assured by checking the sample homogeneity with the computational anatomy toolbox (CAT12) (https://neuro–jena.github.io/cat/) using non-smoothed data, which did not return errors in the registration procedure in any subject. Finally, images were spatially smoothed with an 8-mm full-width at half maximum Gaussian kernel. Total intracranial volume (TIV) was computed by summing the segmented GM, white matter and CSF volumes for each individual.

### PET data acquisition and pre-processing

PET scans were acquired in a Siemens Biograph mCT scanner. Aβ PET images were acquired 90 min post-injection using [^18^F]-flutemetamol with four frames of 5 min each. Afterwards, individual PET frames were co-registered to produce a mean image, which was spatially registered onto the respective structural MRI scan and normalized to the MNI space. We calculated the standardized uptake value ratio (SUVR) in MNI space using the whole cerebellum as the reference region. FDG PET scans were acquired 45 min after the administration of 185 MBq of [^18^F]FDG, and images were reconstructed using a OSEM3D algorithm (8 iterations and 21 subsets) with point spread function (PSF) + time of flight (TOF) corrections. [^18^F]FDG scans were co-registered with the corresponding T1-weighted MRI scan and normalized to the standard MNI space using the transformations calculated for the MRI scan using the DARTEL procedure. [^18^F]FDG uptake in the brain was normalized to the cerebellar vermis.^35^

Prior to statistical analysis, spatially normalized PET images were smoothed with an 8-mm full-width at half maximum Gaussian kernel.

### Definition of the regions of interest

For each image modality, we extracted participants’ values in *a priori* defined regions of interest (ROIs), which were derived from the SPM Anatomy toolbox (https://www.fz-juelich.de/en/inm/inm-7/resources/jubrain-anatomy-toolbox). Aβ PET data were extracted from four different composite ROIs defining the sequential staging of cerebral Aβ accumulation as described in the study by Collij *et al*.^36^ For GMV, data were extracted from composite ROIs identifying regions of early atrophy, including medial and inferior temporal areas. To provide a sequential staging of putative effects of CSF p-tau/Aβ42 ratio, we opted for extracting the GMV data from regions defining the sequential staging of tau pathology as described in the study by Braak and Braak.^37^ Finally, FDG-PET SUVRs were extracted from a composite ROI including the bilateral angular gyrus, the posterior cingulate gyrus and the inferior/middle temporal gyri, previously described as brain areas sensitive to the earliest cortical metabolic failure in Alzheimer’s disease.^38^

### Neuropsychological assessment

We evaluated four cognitive domains, including episodic memory (EM), working memory, abstract reasoning (AR) and cognitive processing speed, for which we have delineated the underlying cerebral morphology in a sub-sample of the present study.^39^ EM was assessed with the immediate and delayed total free and paired recall of the Spanish-adapted version of the Memory Binding Test,^40^ an instrument that was developed for the detection of subtle EM impairments in the cognitively intact population. Working memory, AR and cognitive processing speed were assessed with the Digit Span, the Matrix Reasoning and the coding subtest of the Wechsler Adult Intelligence Scale-Fourth Edition,^41^ respectively. A description of the administration procedure is available in the online Supplementary material.

### Statistical analyses

The impact of the CSF p-tau/Aβ42 ratio on neuroimaging features was assessed both in *a priori* defined ROIs and the whole brain, using voxel-wise methods. In the former approach, we set up a general linear model in R (v. 4.2.3), where the extracted Aβ PET-SUVR, GMV and FDG-SUVR values from the respective ROIs were entered as dependent variables in separate models, while continuous CSF p-tau/Aβ42, age, sex, *APOE*-ɛ4 and years of education were entered as predictors. *APOE*-ɛ4 was coded as binary variable, with 0 denoting non-carriers and 1 carriers of the risk allele. For the analyses assessing GMV, TIV was additionally entered as covariate. Interactions with Alzheimer’s disease risk factors were tested in separate models, by adding an interaction term, as follows:


$$\begin{array}{rl} & Y=\mathrm{CSF}\frac{\mathrm{pTau}}{A\beta 42}+\mathrm{age}+\mathrm{sex}+APOE\varepsilon 4+\mathrm{Edu}\\ & \;+\mathrm{CSF}\;\mathrm{pTau}/A\beta 42\;*\;\mathrm{age} \end{array}$$

$$\begin{array}{rl} & Y=\mathrm{CSF}\frac{\mathrm{pTau}}{A\beta 42}+\mathrm{age}+\mathrm{sex}+APOE\varepsilon 4+\mathrm{Edu}\\ & \;+\mathrm{CSF}\;\mathrm{pTau}/A\beta 42\;*\;APOE\varepsilon 4 \end{array}$$

$$\begin{array}{rl} & Y=\mathrm{CSF}\frac{\mathrm{pTau}}{A\beta 42}+\mathrm{age}+\mathrm{sex}+APOE\varepsilon 4+\mathrm{Edu}\\ & \;+\mathrm{CSF}\;\mathrm{pTau}/A\beta 42\;*\;\mathrm{sex} \end{array}$$

With *Y* denoting GMV, FDG-PET or Aβ PET data. Age was treated as continuous variable in all models. To avoid multicollinearity, continuous p-tau/Aβ42 values were centred to the group mean.^42^ A statistical threshold of two-tailed *P* < 0.05 was selected.

For GMV and FDG-SUVR, an interaction with AT pathophysiology status was additionally inspected, as follows:


$$\begin{array}{rl} & Y=CSF\frac{\mathrm{pTau}}{A\beta 42}+\mathrm{AT}+\mathrm{age}+\mathrm{sex}+APOE\varepsilon 4+\mathrm{Edu}\\ & \;+\mathrm{CSF}\;\mathrm{pTau}/A\beta 42\;*\;\mathrm{AT} \end{array}$$

AT denotes the three-level factor identifying stages of pathology (i.e. A−T−, A+T− and A+T+). To confirm and extend ROI analyses, we performed whole-brain analyses by setting up voxel-wise linear regressions in SPM12 testing both main and interaction effects as described above, where the normalized and smoothed Aβ-PET, FDG-PET and GMV images were entered as dependent variable in separate models, while including the same predictors already included in the ROI analyses. Results were considered significant if surviving a voxel-wise height threshold of *P* < 0.005 with a cluster extent correction of 50 voxels. Finally, main effects of CSF p-tau/Aβ42 and its interaction with risk factors were assessed on cognitive variables, using the same rationale as for the models described above for the ROI analyses.

### Ethics approvals and consent to participate

The ALFA+ study (ALFA-FPM-0311) was approved by the Independent Ethics Committee ‘Parc de Salut Mar’, Barcelona, and registered at Clinicaltrials.gov (Identifier: NCT02485730). All participating subjects signed the study’s informed consent form that had also been approved by the Independent Ethics Committee ‘Parc de Salut Mar’, Barcelona.

## Results

### Sample characteristics

Table 1 summarizes the sample’s demographic information along with cognitive scores, stratified across the AT stages grouping factor. The three groups did not differ in their cognitive performance, total brain volume or educational attainment. The A+T+ group was significantly older than both the A−T− (*post hoc P*-value = 0.02) and the A+T− (*post hoc P*-value = 0.01) groups. Sex and *APOE*-ɛ4 were differently distributed across the three groups, with a greater percentage of males in the A+T− group and significantly more representation of *APOE*-ɛ4 carriers in the A+T− and A+T+ groups. Aβ PET was available for 213 study participants. Supplementary Table 1 shows demographics and cognitive data for this sub-sample.

**Table 1:** Sample characteristics

	A−T−	A+T−	A+T+	Total
*n*	106	113	15	234
Age, years	61.21 (4.51)	61.02 (5.04)	64.82 (4.01)	61.34 (4.81)^a^
Sex, female	76 (71.69%)	58 (51.32%)	9 (60%)	143 (61.11%)^a^
*APOE*-ɛ4	53 (50%)	82 (72.56%)	10 (66.5%)	145 (62%)^a^
CSF Aβ42, pg/ml	1375.31 (167.02)	855.02 (173.57)	844.29 (146.64)	1090.01 (309.91)^a^
CSF p-tau, pg/ml	13.21 (2.82)	12.98 (4.46)	31.1 (6.5)	14.25 (5.94)^a^
CSF p-tau/Aβ42	9.71 (2.24)	16.09 (7.22)	37.63 (8.76)	14.58 (8.84)^a^
Education, years	13.29 (3.5)	13.95 (3.52)	12.67 (3.68)	13.57 (3.53)
TIV, mm^3a^	1397.82 (141.36)	1449.02 (152.86)	1357.42 (183.98)	1419.95 (152.17)
TPR^b^	25.37 (4.27)	24.65 (3.89)	24.62 (3.44)	24.97 (4.04)
TFR^b^	17.79 (5.44)	17.55 (4.88)	16.53 (4.45)	17.59 (5.11)
TDPR^b^	24.62 (4.85)	24.42 (4.19)	24.47 (3.62)	24.51 (4.46)
TDFR^b^	17.45 (5.68)	17.2 (4.98)	16.53 (3.76)	17.27 (5.23)
CPS^b^	65.06 (14.35)	67.11 (15.5)	61.63 (17.84)	65.82 (15.15)
WM^b^	25.21 (5.98)	25.62 (4.87)	24.21 (5.92)	25.33 (5.45)
AR^b^	16.86 (4.55)	16.55 (4.04)	14.93 (5.74)	16.59 (4.41)

Data are presented as mean (SD) or *n* (%).

CPS, cognitive processing speed; TPR, total paired recall; TDPR, total delayed paired recall; TFR, total free recall; TDFR, total delayed free recall; WM, working memory.

^a^Analyses corrected for age and sex.

^b^Analyses corrected for age, sex, years of education and TIV.

### Main effects of CSF p-tau/amyloid-β 42 ratio

**Figure 1:**
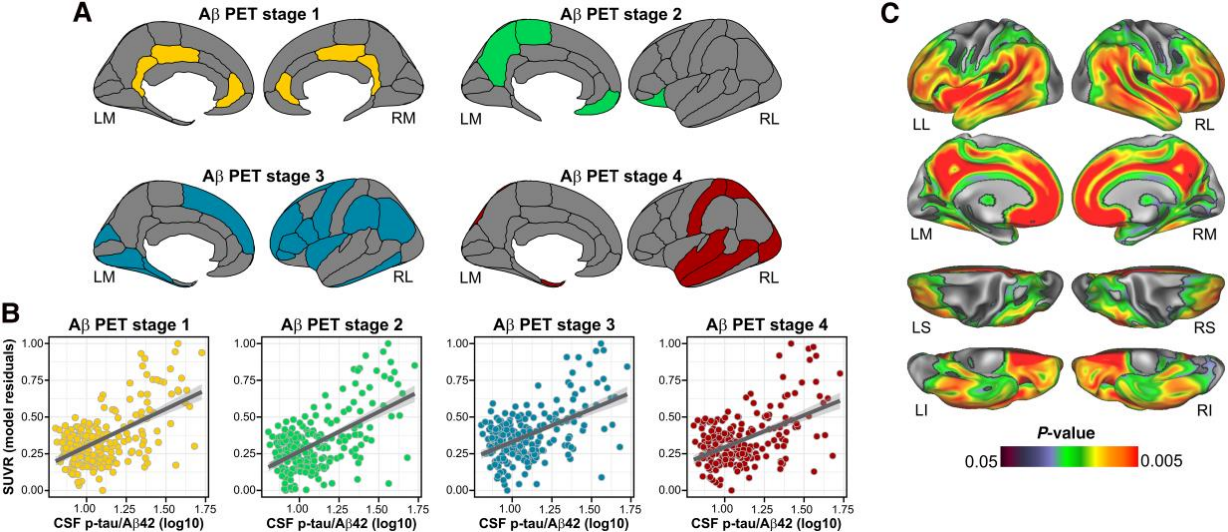
**CSF p-tau/Aβ42 and Aβ deposition. Main effects of CSF p-tau/Aβ42 on fibrillar Aβ deposition.** (**A**) Surface representation of the four composite ROIs of cortical Aβ deposition as in the study by Collij *et al*.^36^ (**B**) Scatterplots showing the significant linear positive associations between CSF p-tau/Aβ42 and Aβ SUVR in *a priori* defined ROIs. Each data point represents an individual subject (*n* = 221). Statistical analyses were performed using linear regression, with the following *F*-values for each stage: *F* = 144.51 for Aβ PET Stage 1, *F* = 145.84 for Aβ PET Stage 2, *F* = 104.54for Aβ PET Stage 3 and *F* = 96.76 for Aβ PET Stage 4. *P*-values were all significant at pFDR < 0.05. (**C**) Surface rendering of the statistical probability maps indicating higher fibrillar Aβ deposition, indicated by a higher SUVR), as a function of CSF p-tau/Aβ42 continuous levels. Statistical analyses were performed using linear regression, and the projected *P*-values correspond to their respective *F*-test. LL, left lateral; LM, left medial; RL, right lateral; RM, right medial.

**Figure 2:**
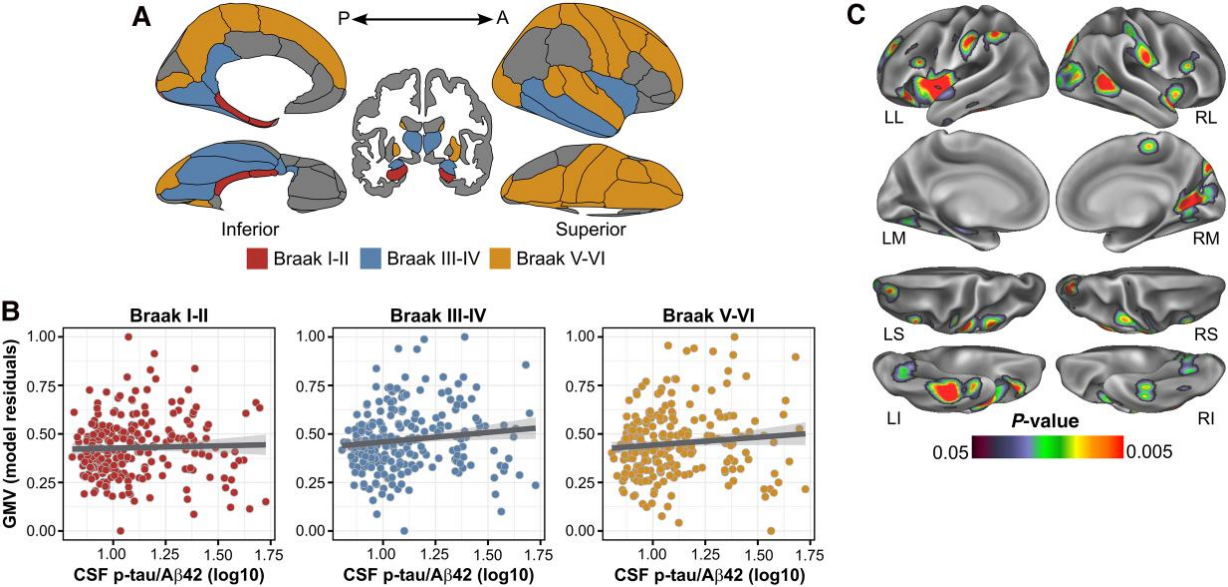
**CSF p-tau/Aβ42 and GMV. Main effects of CSF p-tau/Aβ42 on GMV.** (**A**) Surface representation of the three composite ROIs as defined in Braak and Braak.^37^ (**B**) Scatterplots showing the significant linear positive associations between CSF p-tau/Aβ42 and GMV in *a priori* defined ROIs. Each data point represents an individual subject (*n* = 234). Statistical analyses were performed using linear regression, with the following *F*-values for each stage: *F* = 0.28 for Braak I and II, *F* = 4.31 for Braak III and IV and *F* = 2.61 for Braak V and VI. *P*-value at Braak III and IV was significant at uncorrected *P* < 0.05. (**C**) Surface rendering of the statistical probability maps indicating a greater GMV as a function of CSF p-tau/Aβ42 continuous levels. Statistical analyses were performed using linear regression, and the projected *P*-values correspond to their respective *F*-test. LL, left lateral; LM, left medial; RL, right lateral; RM, right medial.

We found a significantly positive association between CSF p-tau/Aβ42 and Aβ SUVR in all four Aβ stages ROIs (Stage 1: *F*_1212_ = 144.51, *P* < 0.001, false discovery rate *P*-value (pFDR) < 0.05, $\eta_{p}^{2}=0.41$; Stage 2: *F*_1212_ = 145.84, *P* < 0.001, pFDR < 0.05, $\eta_{p}^{2}=0.42$; Stage 3: *F*_1212_ = 104.54, *P* < 0.001, pFDR < 0.05, $\eta_{p}^{2}=0.33$ and Stage 4: *F*_1212_ = 96.76, pFDR < 0.05, *P* < 0.001, $\eta_{p}^{2}=0.31$; Fig. 1A and B). On the whole brain, this association was significant in widespread regions including medial frontal, lateral temporal and midline cortical areas (Fig. 1C). When inspecting GMV, we found a nominally significant main effect of CSF p-tau/Aβ42 in the Braak III and IV ROI indicating a positive association, which, however, did not survive correction for multiple testing (*F*_1228_ = 4.03, *P* = 0.04, pFDR = 0.11, $\eta_{p}^{2}=0.31$; Fig. 2A and B). On the whole-brain level, this association additionally included the inferior temporal gyrus, insula, caudate and the right middle temporal gyrus, bilaterally (Fig. 2C). No significant associations were observed when inspecting FDG-SUVR in either the ROI or whole-brain analyses. The inclusion of the time interval between CSF data acquisition and PET or MRI as a covariate did not significantly alter the results (data not shown).

Supplementary Table 2 shows the direct comparison of the *F*-statistics and effect size ($\eta_{p}^{2}$) between CSF p-tau/Aβ42 and the other two core Alzheimer’s disease reference biomarkers (i.e. CSF Aβ42 and CSF p-tau). Supplementary Table 3 shows statistical results of the whole-brain analyses for the main effects of CSF p-tau/Aβ42 on GMV. For the main effects on Aβ SUVR, our analyses yielded only one cluster, reported in Fig. 1C.

### Interaction with Alzheimer’s disease risk factors

**Figure 3:**
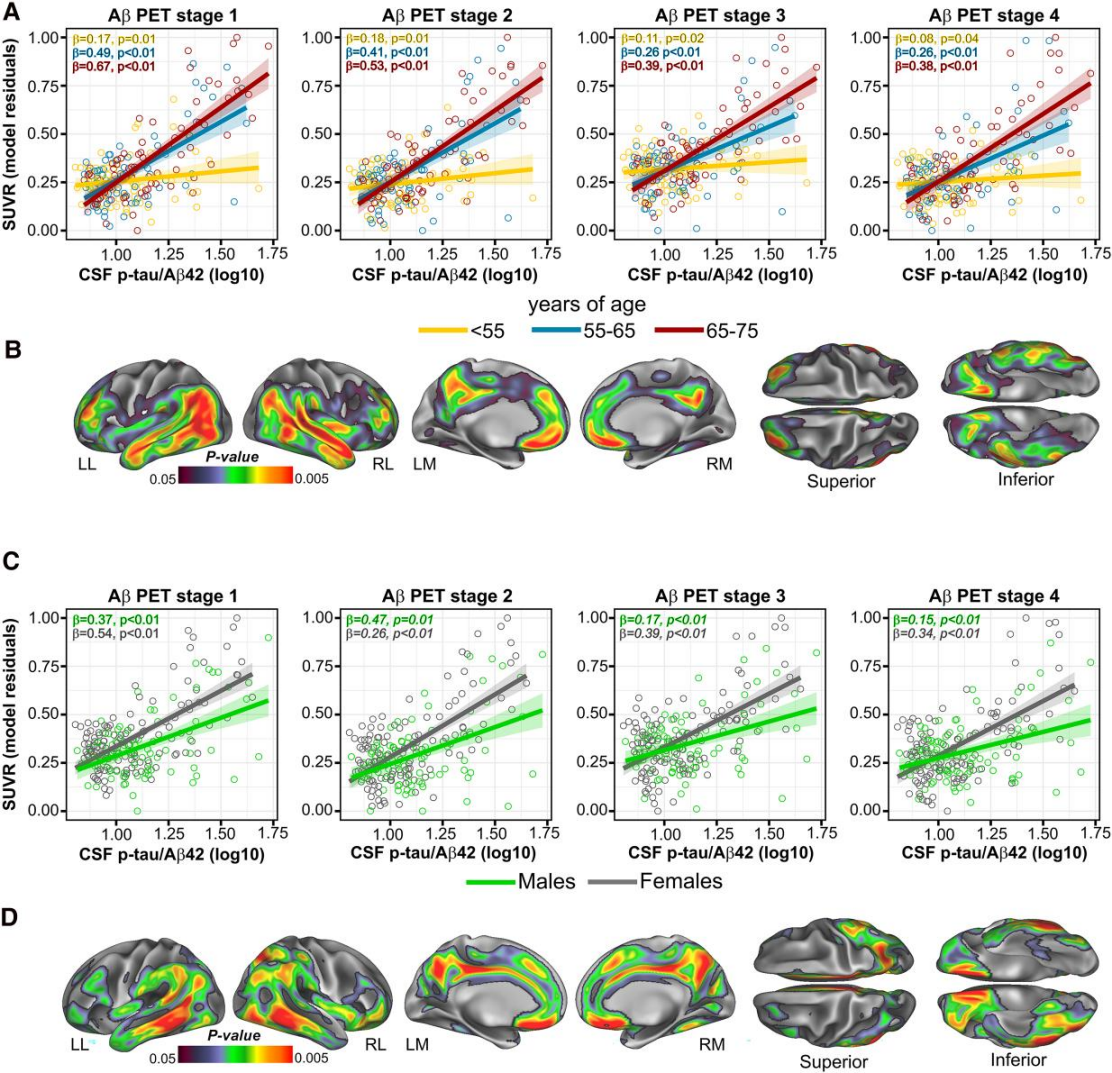
**CSF p-tau/Aβ42 interactions with age and sex on Aβ deposition.** Older age and female sex are associated with a higher Aβ deposition for a given CSF p-tau/Aβ42 level. (**A**) Group scatterplots showing the significant interactions between continuous CSF p-tau/Aβ42 and age on Aβ SUVR in the *a priori* defined ROIs. For visualization purposes, age was broken down into three groups using tercile ranking. In each scatterplot, parameter estimates and *P*-values are provided for each age subgroup. . Each data point represents an individual subject (*n* = 221). Statistical analyses were performed using linear regression, with the following *F*-values for each stage: *F* = 13.98 for Aβ PET Stage 1, *F* = 18.32 for Aβ PET Stage 2, *F* = 18.14 for Aβ PET Stage 3 and *F* = 19.86, for Aβ PET Stage 4. *P*-values were all significant at pFDR < 0.05. (**B**) Surface rendering of the statistical probability maps showing the significant interaction between CSF p-tau/Aβ42 and age, on the whole-brain level. Statistical analyses were performed using linear regression, and the projected *P*-values correspond to their respective *F*-test. (**C**) Group scatterplots showing the significant interactions between continuous CSF p-tau/Aβ42 and sex in the *a priori* defined ROIs. In each scatterplot, parameter estimates and *P*-values are provided for males and females. Each data point represents an individual subject (*n* = 221). Statistical analyses were performed using linear regression, with the following *F*-values for each stage: *F* = 6.25 for Aβ PET Stage 1, *F* = 10.55 for Aβ PET Stage 2, *F* = 9.69 for Aβ PET Stage 3 and *F* = 11.54 for Aβ PET Stage 4. *P*-values were all significant at pFDR < 0.05. (**D**) Surface rendering of the statistical probability maps showing the significant interaction between CSF p-tau/Aβ42 and sex, on the whole-brain level. Statistical analyses were performed using linear regression, and the projected *P*-values correspond to their respective *F*-test. LL, left lateral; LM, left medial; RL, right lateral; RM, right medial.

**Figure 4:**
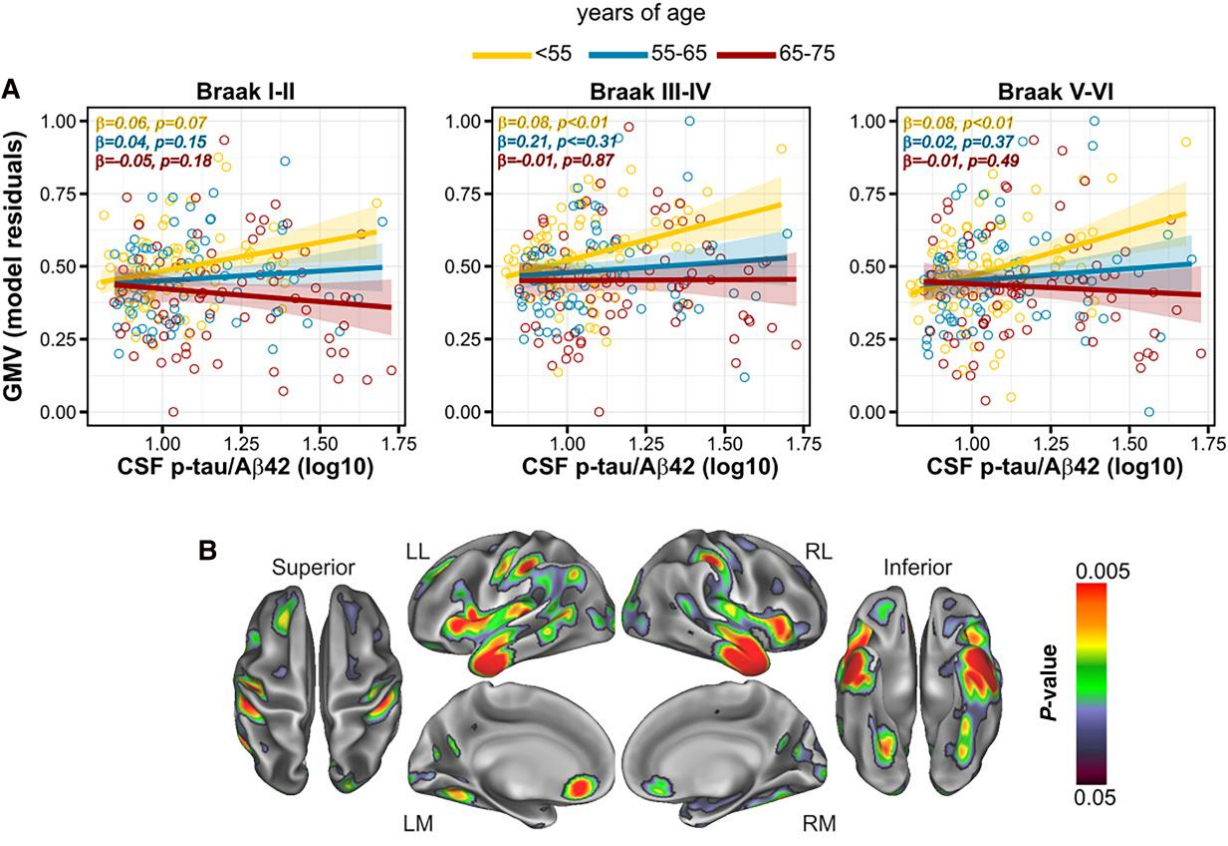
**CSF p-tau/Aβ42 interactions with age on GMV.** Younger age was associated with a greater GMV for a given CSF p-tau/Aβ42 level. (**A**) Group scatterplots showing the significant interactions between continuous CSF p-tau/Aβ42 and age on GMV, in the *a priori* defined ROIs. For visualization purposes, age was broken down into three groups using tercile ranking. In each scatterplot, parameter estimates and *P*-values are provided for each age subgroup. Each data point represents an individual subject (*n* = 234). Statistical analyses were performed using linear regression, with an *F*-value = 4.21 for the Braak V and VI stage, which was significant on a nominal level, *P* < 0.05. (**B**) Surface rendering of the statistical probability maps showing the significant interaction between CSF p-tau/Aβ42 and age on GMV, on the whole-brain level. Statistical analyses were performed using linear regression, and the projected *P*-values correspond to their respective *F*-test.

When looking at Aβ SUVR we found significant interactions between CSF p-tau/Aβ42 and age in all four Aβ stages ROI, indicating that the effect of the CSF biomarker was significantly larger with more advanced age (Stage 1: *F*_6213_ = 13.98, *P* < 0.001, pFDR < 0.05, $\eta_{p}^{2}=0.06$; Stage 2: *F*_6213_ = 18.32, *P* < 0.001, pFDR < 0.05, $\eta_{p}^{2}=0.08$; Stage 3: *F*_6213_ = 18.14, *P* < 0.001, pFDR < 0.05, $\eta_{p}^{2}=0.08$ and Stage 4: *F*_6213_ = 19.86, *P* < 0.001, pFDR < 0.05, $\eta_{p}^{2}=0.09$; Fig. 3A). Whole-brain analyses confirmed these interactions in bilateral temporo-parietal areas and in the anterior and posterior cingulate cortex (Fig. 3B; Supplementary Table 4). Furthermore, we found a significant interaction between CSF p-tau/Aβ42 and sex indicating that, for the same level of the CSF biomarker, females showed a significantly higher Aβ PET uptake in all four Aβ stages ROI (Stage 1: *F*_6213_ = 6.25, *P* = 0.01, pFDR < 0.05, $\eta_{p}^{2}=0.03$; Stage 2: F_6213_ = 10.55, *P* = 0.001, pFDR < 0.05, $\eta_{p}^{2}=0.05$; Stage 3: *F*_6213_ = 9.69, *P* = 0.02, pFDR < 0.05, $\eta_{p}^{2}=0.04$ and Stage 4: *F*_6213_ = 11.54, *P* < 0.001, pFDR < 0.05, $\eta_{p}^{2}=0.09$; Fig. 3C). Whole-brain analyses confirmed these interactions in bilateral middle temporal areas, fusiform, the anterior and posterior cingulate cortex (Fig. 3D; Supplementary Table 5). No significant interactions were found between CSF p-tau/Aβ42 and *APOE*-ɛ4 in any ROIs; however, the whole-brain analysis revealed a significant interaction in the left hippocampus [*t*_206_ = 3.82, *P* < 0.001, cluster size (*k*) = 141 (*x* = −30, *y* = −14, *z* = −24)], indicating that *APOE*-ɛ4 carriers compared with non-carriers had a significantly higher Aβ fibrillar deposition for a given concentration of the CSF biomarker (Supplementary Fig. 1 and Table 6). When assessing GMV, we found a nominally significant interaction between CSF p-tau/Aβ42 and age in the Braak V/VI composite ROI, which, however, did not survive correction for multiple testing (*F*_1226_ = 4.21, *P* = 0.04, pFDR = 0.11, $\eta_{p}^{2}=0.02$), indicating a significantly less positive association between the CSF biomarker and GMV with older age (Fig. 4A). The whole-brain analysis additionally revealed a significant interaction mapping onto the bilateral anterior insula and temporal poles (Fig. 4B; Supplementary Table 7). Finally, an interaction with sex indicated that, for the same level of continuous CSF p-tau/Aβ42, women showed significantly reduced GMV in the right precuneus [*t*_227_ = 3.39, *P* < 0.001, cluster size (*k*) = 350] and the bilateral fusiform gyrus [*t*_227_ = 3.81, *P* < 0.001, cluster size (*k*) = 127; Supplementary Table 8]. This interaction, however, did not reach statistical significance in any of the *a priori* defined ROIs. No significant interactions with any of the assessed Alzheimer’s disease risk factors were found for FDG-SUVR. The inclusion of the time interval between CSF data acquisition and PET or MRI as a covariate did not significantly alter the results (data not shown).

### Interaction with AT staging factor

**Figure 5:**
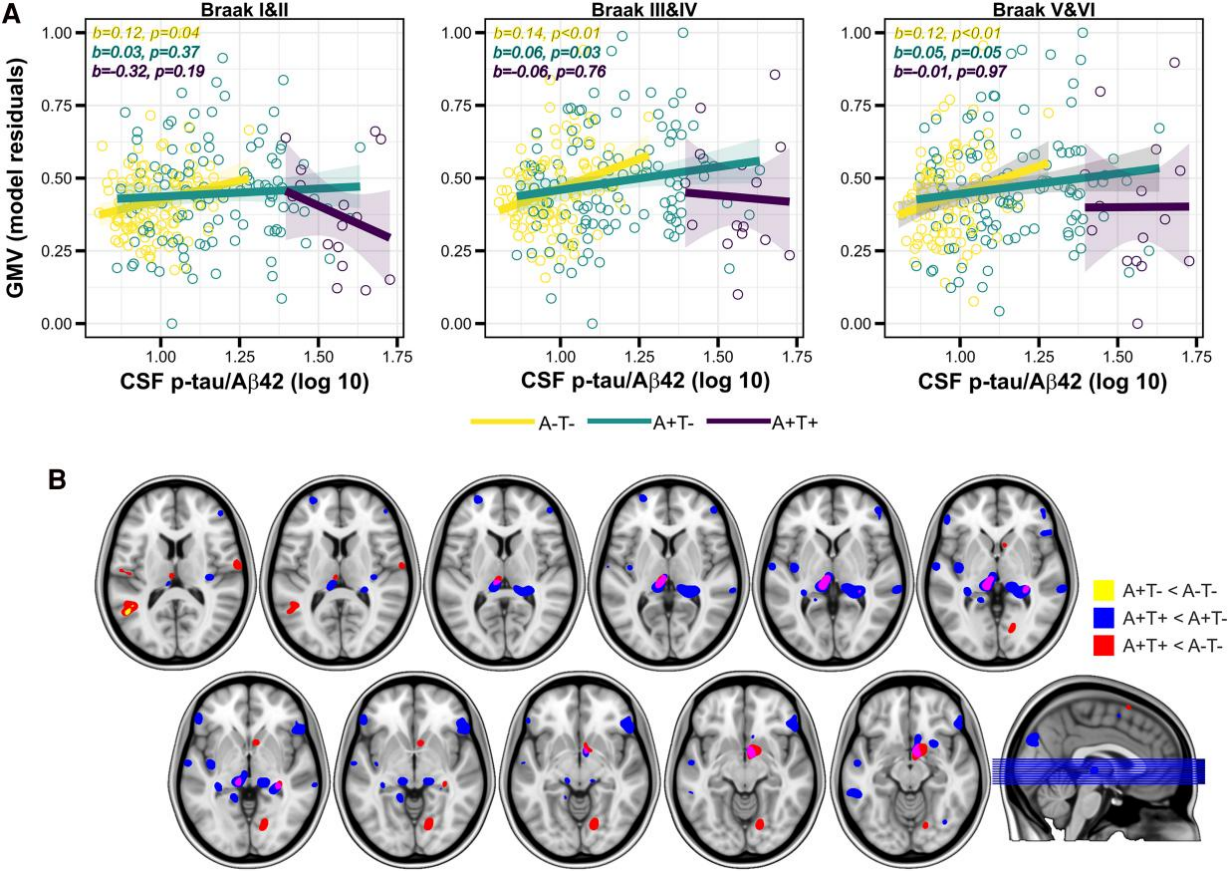
**CSF p-tau/Aβ42 interaction with Aβ status on GMV.** The impact of CSF p-tau/Aβ42 ratio on GMV was significantly modulated by Aβ status. (**A**) Group scatterplots showing the linear association between CSF p-tau/Aβ42 ratio and GMV in three Braak composite ROIs. For visualization purposes, linear slopes are broken down by the three AT stage groups. In each scatterplot, parameter estimates and *P*-values are provided for each age subgroup. Each data point represents an individual subject (in = 234). Statistical analyses were performed using linear regression, which yielded with non-significant *F*-values. By contrast, the interaction with binary category Aβ status was significant in Braak III and IV and Braak V and VI for uncorrected *P* < 0.05. (**B**) Volume rendering of GMV clusters of paired comparisons between AT pathophysiology grouping factors.

We found no significant interactions between continuous CSF p-tau/Aβ42 ratio and AT staging factor in any of the assessed ROIs. Due to the relative small number of A+T+ individuals (*n* = 15, 6.41% of the entire sample), we ran interactions with Aβ status, calculated on CSF Aβ42 values (see the ‘CSF sampling and analysis’ section). These analyses revealed nominally significant interactions in the Braak III and IV and Braak V and VI composite ROIs, while for Braak I and II, the interaction just missed significance (Braak I and II: *F*_1225_ = 3.59, *P* = 0.05, pFDR = 0.12, $\eta_{p}^{2}=0.02$; Braak III and IV: *F*_1225_ = 4.77, *P* = 0.03, pFDR = 0.09, $\eta_{p}^{2}=0.02$ and Braak V and VI: *F*_1225_ = 4.08, *P* = 0.04, pFDR = 0.09, $\eta_{p}^{2}=0.02$). This indicates that the impact of the CSF p-tau/Aβ42 ratio on regional GMV was significantly affected by Aβ status. For visualization purposes, Fig. 5A and B shows the linear associations between CSF p-tau/Aβ42 ratio and pathophysiological stages, broken down into AT subgroups. When assessing brain metabolism, we found that, for the same level of CSF p-tau/Aβ42 ratio, the A+T+ showed an increased FDG-PET signal in the bilateral posterior cingulate gyrus, compared with the A−T− group [*t*_226_ = 3.97, *P* < 0.001, cluster size (*k*) = 76; data not shown].

### Impact on cognitive performance

**Figure 6:**
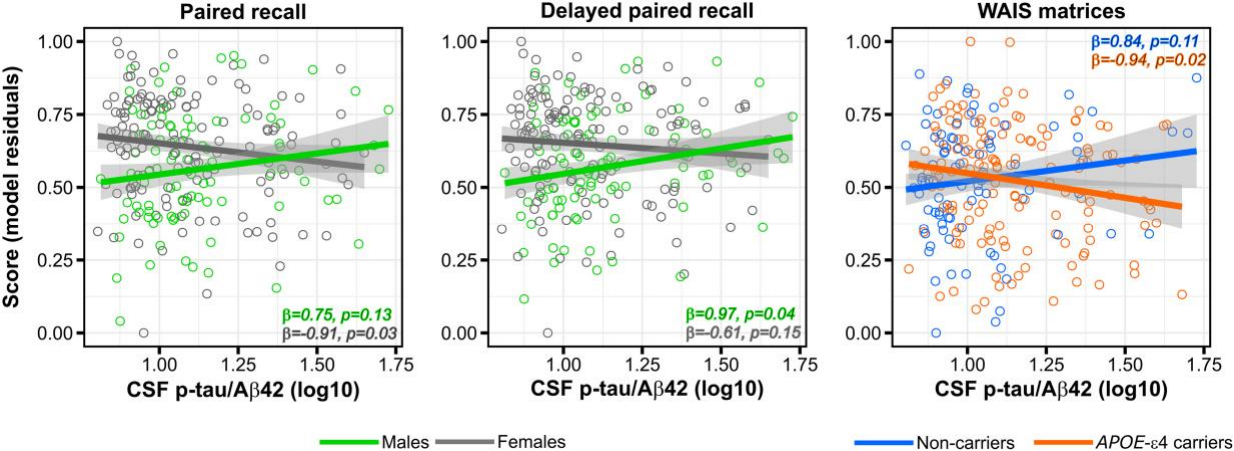
**CSF p-tau/Aβ42 interactions with *APOE*-ɛ4 on cognitive performance.** The association between CSF p-tau/Aβ42 and cognitive performance was modulated by both sex and *APOE*-ɛ4. Group scatterplots showing that, for the same level of CSF p-tau/Aβ42, women compared with men had a poorer cognitive performance in immediate and delayed paired recall, while *APOE*-ɛ4 carriers compared with non-carriers had a poorer performance in AR. Each data point represents an individual subject (*n* = 234). Statistical analyses were performed using linear regression, with *F*-values being *F* = 4.98 for immediate recall, *F* = 4.42 for delayed recall and *F* = 5.32 for AR. All *P*-values were significant at uncorrected *P* < 0.05.

There were no significant associations between CSF p-tau/Aβ42 and any measure of cognitive performance. However, we observed significant interactions with sex on two Memory Binding Test subdomains, indicating that the impact of CSF p-tau/Aβ42 ratio levels on both immediate (total paired recall: *F*_1225_ = 4.98, *P* = 0.02, $\eta_{p}^{2}=0.02$) and delayed (total delayed paired recall: *F*_1225_ = 4.42, *P* = 0.04, $\eta_{p}^{2}=0.02$) paired recall was significantly modulated by sex, with women showing poorer performance for the same level as the CSF biomarker, compared with men. In addition, we found that *APOE*-ɛ4 modified the association between CSF p-tau/Aβ42 and AR, indicating a significantly more negative association in ɛ4-carriers compared with non-carriers (*F*_1225_ = 5.32, *P* = 0.02, $\eta_{p}^{2}=0.02$; Fig. 6). The inclusion of the time interval between CSF and cognitive data acquisition as a covariate did not significantly alter the results (data not shown).

## Discussion

In the present work, we report that the CSF p-tau/Aβ42 ratio is significantly associated with fibrillary Aβ deposition and brain morphology in a sample of middle-aged CU individuals at the earliest inception of pre-clinical Alzheimer’s *continuum*. These associations were modulated by unmodifiable risk factors for Alzheimer’s disease, including older age, female sex and the *APOE*-ɛ4 genotype. As for the main effects, we have shown a positive association between CSF p-tau/Aβ42 ratio and cortical Aβ deposition in all four ROIs identifying the stages of Aβ accumulation. The whole-brain analysis confirmed this significant association in widespread areas and revealed other areas of Aβ deposition as a function of the CSF biomarker, such as the bilateral thalamus, striatum, and more extended areas of the middle cingulate cortex. Furthermore, we observed, although only at a nominal level of significance, a positive association between continuous CSF p-tau/Aβ42 and GMV in the Braak III and IV composite ROI, which includes the inferior temporal, orbitofrontal and posterior midline cortices.

Our observation of a concomitant effect of the CSF p-tau/Aβ42 ratio on both fibrillary Aβ deposition and on GMV in regions sensitive to the Alzheimer’s disease pathology in asymptomatic individuals suggests that this hybrid marker may be sensitive in predicting the clinical trajectory, as already observed in samples at more advanced stages.^22-25^

It is worth noting that the spatial maps and significance values we report here are more widespread than in our previous work, where we analysed the relationship between soluble and fibrillar Aβ, using the CSF Aβ42/40 as a surrogate marker of Aβ dysmetabolism,^43^ of which this study constitutes a sub-sample. Moreover, the larger effect sizes we report in comparison with both CSF Aβ42 and p-tau suggest a superior performance of CSF p-tau/Aβ42 in its association with Aβ deposition (i.e. Aβ PET) and GMV.

This is in agreement with the evidence reported earlier that the CSF p-tau/Aβ42 ratio outperforms Aβ42 in predicting cerebral Aβ deposition.^22-25^ Collectively, these findings foster the use of the p-tau/Aβ42 ratio as the preferred measure in clinical practice and trials.

We then look for interactions with Alzheimer’s disease risk actors and found that this association was significantly modulated by age, where older individuals exhibited higher Aβ accumulation compared with younger ones, for the same level of CSF p-tau/Aβ42 ratio. The whole-brain analysis confirmed these results and further revealed a significant interaction effect in the striatum. Ageing is intrinsically associated with multiple biological failures, encompassing diverse pathophysiological processes among which are an increased blood–brain barrier permeability, neuroinflammation, synaptic and vascular dysfunction.^44^ These age-related changes can exacerbate Aβ accumulation in the presence of protein dysmetabolism captured in CSF concentrations. In addition to age, we reported that *APOE*-ɛ4 carriers exhibited a greater Aβ deposition in the hippocampus compared with non-carriers, for a given CSF p-tau/Aβ42 level. This is in striking agreement with our previously reported data on the modulatory role of *APOE* status in driving fibrillary Aβ deposition as a function of early Aβ dysmetabolism in medial temporal lobe areas.^43^ Given that the medial temporal lobe (MTL) is particularly vulnerable to early tau rather than Aβ deposition,^45^ our findings here reinforce the idea that *APOE*-ɛ4 may drive a synergistic interaction between Aβ and tau in the earliest Alzheimer’s *continuum.* This may also underline the faster cognitive decline previously documented in asymptomatic carriers of the *APOE*-ɛ4 allele.^46,47^ In line with this, we have reported here a significant interaction indicating that unlike non-carriers*, APOE*-ɛ4 carriers displayed a negative association between the CSF p-tau/Aβ42 ratio and AR, which is also in agreement with our previous observation of a modulatory role of *APOE*-ɛ4 on the association between regional GMV and performance in different cognitive domains.^48^

We found that sex modulated the association between CSF p-tau/Aβ42 and both fibrillar Aβ deposition and GMV, although in different topologies, with the effects on Aβ-PET being more widespread than those on GMV. Female sex is considered a risk factor for Alzheimer’s disease, particularly during the perimenopause, a transitional phase occurring during midlife, characterized by oestrogen depletion with consequent loss of neuroprotective function.^49,50^ Previously, we reported that compared with men, women showed a stronger association between incipient Aβ burden and neurodegeneration indexed by CSF neurofilament light concentrations.^51^ Moreover, an interaction between genetic risk and sex has been documented, indicating that, among the *APOE*-ɛ4 carriers, women present an increased risk to convert from mild cognitive impairment to Alzheimer’s disease.^52^ Our data add further evidence that women may be more exposed to the deleterious effects of incipient Alzheimer’s disease pathology, captured by the CSF p-tau/Aβ42 ratio. This interpretation is further corroborated by our data showing a worse EM in women compared with men, for the same level of the CSF biomarker.

The interaction with sex in driving GMV variations mapped onto the precuneus and fusiform areas. Structural integrity in the precuneus plays a major role in preserved memory functions,^53,54^ and indeed, we reported that, unlike in males, a higher concentration of CSF p-tau/Aβ42 was related to worse episodic recall in female participants.

As for the observed effect sizes, in line with the recommendations of Cohen,^55^ our results show that for the main effects on both fibrillary Aβ deposition (SUVR) and brain morphology (GMV), we obtained large effect sizes. This reflects a robust association between the p-tau/Aβ42 ratio and these neuropathological markers, suggesting that this biomarker effectively captures the early brain alterations associated with Alzheimer’s disease, even in cognitively normal individuals. By contrast, the medium effect sizes found in the interactions with the assessed risk factors suggest that, although these factors modulate the relationships between CSF p-tau/Aβ42 and neurodegenerative markers, their impact is moderate compared with the direct effects of the biomarker itself. This moderate magnitude of the interaction effects may align with the complexity of the interactions among genetic, biological and environmental factors that modulate disease progression in older age.

As noted above, we found a significantly larger GMV in Braak III/IV composite ROI as a function of CSF p-tau/Aβ42 ratio, a finding that was extended to a symmetric pattern of brain areas including the inferior temporal gyrus, insula, caudate and the right middle temporal gyrus, in the voxel-wise analyses. Previous studies have reported increased GMV and/or cortical thickness as a function of Aβ deposition in CU individuals,^3-6^ but no study has so far reported these increments as a function the CSF p-tau/Aβ42 ratio. Higher GMV as a function of Aβ pathology has been interpreted as a brain structural correlate of neuroinflammatory processes,^6,56^ possibly due to Aβ-driven glial activation^57^ or attributed to compensatory neuroplasticity, increased neural activity or neural hypertrophy.^58,59^ In our previous work conducted in the same study cohort, we reported that as soon as the CSF p-tau/Aβ42 ratio status becomes positive, there was a steep increase of different downstream markers of Alzheimer’s disease pathology, including glial markers such as glial fibrillary acidic protein, YKL-40 and soluble triggering receptor expressed on myeloid cells 2.^60^ Additionally, we reported that fibrillary Aβ deposition, indexed by a higher Centiloid value, was positively associated with CSF glial markers.^51^ In line with this and based on the findings of the present study, it could be speculated that an initial glial response as a function of the earliest Alzheimer’s disease pathophysiological alteration captured by the CSF p-tau/Aβ42 ratio may underlie an initial regional GMV hypertrophy. However, as individuals proceed in the pathophysiological *continuum*, such GMV increments may transition towards neurodegeneration. In support of this, we observed that the association between the CSF biomarker and GMV was significantly modified by Aβ status in widespread cortical areas (i.e. Braak III/IV and Braak V/VI) and that overall GMV as a function of the CSF p-tau/Aβ42 ratio decreases along the more severe stages of pathophysiology (Fig. 5A). However, we must acknowledge that the interaction with the three-level AT status factor was not significant in our analyses, only the interaction with Aβ status. This likely occurs due to a limitation of our sample characteristics, which includes only *n* = 15 participants (6.41%) with an A+T+ profile. Future studies should employ more balanced samples across the AT stages.

Moreover, our interpretation of the results observed for both the main effects and interactions concerning GMV should be taken with caution, as the significance values were only nominal and did not survive correction for multiple testing. Nonetheless, we must emphasize that this study has a primarily exploratory nature, and given that our subjects are CU, we do not expect to observe large effect sizes in relation to brain morphology.

A strength of the present study is that it used a sample composed of middle-aged CU individuals, with 45% being negative to Aβ CSF markers (i.e. CSF Aβ42), which allows studying the brain structural and functional correlates of Alzheimer’s disease pathology at the earliest disease inception. Another strength is the availability of a multi-modal neuroimaging dataset, which provides a comprehensive picture of multiple downstream neuropathological events as a function of the incipient Alzheimer’s disease pathology. However, a limitation of our work is represented by its cross-sectional nature, which prevents us from establishing any causal inference of the observed associations or determining their prospective course over time. Additionally, the cross-sectional design limits our ability to assess cognitive differences with regard to each participant’s position relative to their ‘peak’ cognitive level. Without prior information, we cannot ascertain how close or far individuals are from their optimal cognitive performance at the time of assessment. While we have used education as a proxy to mitigate this limitation as far as possible, this remains an important consideration. Further insights are forthcoming as follow-up data in the ALFA+ cohort are currently being gathered. Another limitation of our study is that we used only one neuropsychological test per cognitive domain. This may not fully capture the complexity of cognitive functions, potentially missing nuances in cognitive performance that could be revealed through a broader battery of tests.

## Conclusion

In summary, we report that the biomarker CSF p-tau/Aβ42 ratio is strongly associated with fibrillary Aβ deposition and with increased GMV in CU individuals at the earliest inception of the Alzheimer’s *continuum*. The impact of the CSF ratio on both Aβ deposition and brain structure is modulated by specific risk factors, with this highlighting the need to take into account such predisposing variables in both clinical practice and prevention trials.

## Supplementary Material

fcae451_Supplementary_Data

## Data Availability

The data that support the findings of this study are available from the corresponding authors, upon reasonable request.

## Appendix I

Collaborators of the ALFA study are as follows: Ricardo A. Aguilar, Annabella B. Gorriti, Anna B. Serrat, Raffaele Cacciaglia, Lidia C. Gispert, Alba C. Martinez, Marta D. Milan, Carmen D. Gomez, Ruth D. Iglesias, Marie E. F. Karine, Sherezade F. Julian, Patricia G. Serra, Juan D. Gispert, Armand G. Escalante, Oriol G. Rivera, Laura H. Penas, Gema H. Rodriguez, Jordi H. Ninou, Laura I. Gamez, Iva Knezevic, Paula M. Alvarez, Tania M. Diaz, Carolina M. Gil, Eva Palacios, Maria Pascual, Albina P. Ballester, Sandra P. Mendez, Irina A. Radoi, Blanca R. Fernandez, Laura R. Freixedes, Aleix S. Vila, Gonzalo A. Sanchez Benavides, Mahnaz S. Mahnaz, Lluis S. Harster, Anna S. Prat, Laura S. Stankeviciute, Marc S. Calvet, Marc V. Jaramillo, Natalia V. Tejedor, Annabella Beteta, Alba Cañas, Carme Deulofeu, Irene Cumplido, Ruth Dominguez, Maria Emilio, Sherezade Fuentes, Laura Hernandez, Gema Huesa, Jordi Huguet, Paula Marne, Tania Menchón, Albina Polo, Sandra Pradas, Anna Soteras and Marc Vilanova.
