# Supplementary material for: The CSF p-tau/β-amyloid 42 ratio correlates with brain structure and fibrillary β-amyloid deposition in cognitively unimpaired individuals at the earliest stages of pre-clinical Alzheimer’s disease
Source: Brain Commun. 2024 Dec 13;7(1):fcae451. doi: 10.1093/braincomms/fcae451 (PMC11668178; doi:10.1093/braincomms/fcae451)
Supplement: fcae451_Supplementary_Data [file fcae451_supplementary_data.docx]

**Title:** The CSF p-tau/Aβ42 ratio correlates with brain structure and fibrillary Aβ deposition in cognitively unimpaired individuals at the earliest stages of preclinical Alzheimer’s disease

**Authors:** Raffaele Cacciaglia, Mahnaz Shekari, Gemma Salvadó, Marta Milà-Alomà, Grégory Operto, Carles Falcon, Gonzalo Sánchez-Benavides, Carolina Minguillón, Karine Fauria, Oriol Grau-Rivera, José Luis Molinuevo, Kaj Blennow, Henrik Zetterberg, Frances-Catherine Quevenco, Marc Suárez-Calvet, and Juan Domingo Gispert, for the ALFA study.

**Supplementary materials**

**Supplementary Methods**

***Procedures for neuropsychological assessment***

Episodic memory was assessed using the Memory Binding Test (MBT). Previous studies have established the ability of the MBT (formerly referred as Memory Capacity Test [MCT]) to discriminate subjects with cerebral Aβ deposition (Papp et al., 2015), to successfully discriminate individuals with mild cognitive impairment (MCI) from normal elderly subjects (Buschke et al., 2017) and also successfully predict the incidence of MCI longitudinally (Mowrey et al., 2016). The MBT uses a controlled learning procedure to ensure that any recall deficit can be attributed to effective memory impairment and not to deficiency in any other cognitive strategies. During administration, the examinee sequentially learns two lists of 16 words written in cards, where each card contains four words. The lists share semantic categories, which are used both to control the encoding of the words in learning and as cues during cued recall trials. In the present study, we included the two main MBT outcome, namely Total Paired Recall (TPR) and Total Free Recall (TFR). TPR indexes the immediate recall of both lists after semantic cueing, while TFR provides a measure of the immediate free recall, with no sematic cueing, of both lists.

Cognitive processing speed was assessed with the Coding subtest of the Wechsler Adult Intelligence Scale-Fourth Edition (Wechsler, 2012). Here, participants are given keys that match numeric digits spanning from 1 to 9 with a symbol. The task is to write down the correct symbol next to a list of numbers as quickly as possible. Finally, working memory (WM), abstract reasoning (AR) were assessed with the Digit Span and the Matrix Reasoning subscales of the WAIS. For WM assessment, we considered the sum of the Digit Span forward, backward, and sequencing (digit-span total).

**Supplementary** **Table 1 – Demographics and cognitive characteristics of the subsample with available Aβ PET data**

|  |  | **A-T-** |  | **A+T-** |  | **A+T+** |  | **Total** |
| --- | --- | --- | --- | --- | --- | --- | --- | --- |
| **n** |  | 97 |  | 102 |  | 14 |  | 213 |
| **Age, y** |  | 61.27 (4.50) |  | 60.71 (4.92) |  | 64.88 (4.16) |  | 61.23 (4.77)* |
| **Sex, f** |  | 70 (72.16%) |  | 51 (50%) |  | 9 (64.28%) |  | 130 (61.03%)* |
| ***APOE*-ε4** |  | 47 (48.45%) |  | 75 (73.52%) |  | 10 (71.42%) |  | 132 (61.97%)* |
| **CSF Aβ42, pg/ml** |  | 1,365.65 (163.91) |  | 855.85 (171.42) |  | 840.03 (151.21) |  | 1,086.97 (304.68)* |
| **CSF p-Tau, pg/ml** |  | 12.96 (2.67) |  | 13.07 (4.41) |  | 30.10 (5.41) |  | 14.14 (5.68)* |
| **CSF p-Tau/Aβ42** |  | 9.60 (2.28) |  | 16.17 (7.23) |  | 36.75 (8.38) |  | 14.53 (8.75)* |
| **Education, y** |  | 13.38 (3.45) |  | 13.89 (3.53) |  | 12.36 (3.61) |  | 13.56 (3.51) |
| **TIV, mm^3^*** |  | 1,395.93 (145.17) |  | 1,447.50 (153.63) |  | 1,337.16 (172.68) |  | 1,416.76 (153.91) |
| **TPR**** |  | 25.61 (4.18) |  | 25.01 (3.76) |  | 24.64 (3.56) |  | 25.26 (3.94) |
| **TFR**** |  | 18.06 (5.36) |  | 17.81 (4.85) |  | 16.93 (4.34) |  | 17.87 (5.05) |
| **TDPR**** |  | 24.92 (4.65) |  | 24.75 (4.08) |  | 24.50 (3.76) |  | 24.81 (4.32) |
| **TDFR**** |  | 17.73 (5.49) |  | 17.45 (4.83) |  | 16.57 (3.90) |  | 17.52 (5.08) |
| **Coding**** |  | 65.40 (14.09) |  | 67.19 (15.70) |  | 59.93 (17.25) |  | 65.90 (15.13) |
| **Digit Span**** |  | 25.22 (5.99) |  | 25.61 (4.82) |  | 23.57 (5.60) |  | 25.30 (5.43) |
| **Matrix Reasoning**** |  | 16.79 (4.51) |  | 16.62 (4.05) |  | 14.50 (5.69) |  | 16.56 (4.40) |

Data are presented as mean (SD) or n (%); AR: abstract reasoning; CPS: cognitive processing speed; CSF: cerebrospinal fluid; TPR: total paired recall; TDPR: total delayed paired recall; TFR: total free recall; TDFR: total delayed free recall; TIV: total intracranial volume; WM: working memory. *Analyses corrected for age and sex; **Analyses corrected for age, sex, years of education, and total intracranial volume.

**Supplementary Table 2 – Comparison of F-statistics and effect sizes among three different CSF biomarkers (main effects)**

| **Predictor** | **Aβ deposition (Aβ PET)** | | | | **Gray Matter Volume** | | |
| --- | --- | --- | --- | --- | --- | --- | --- |
|  | **Aβ stage 1** | **Aβ stage 2** | **Aβ stage 3** | **Aβ stage 4** | **Braak I&II** | **Braak III&IV** | **Braak V&VI** |
| **CSF Aβ42** | 45.6 (0.18) | 40.9 (0.16) | 26.03 (0.11) | 22.09 (0.09) | 0.75 (<0.01) | 2.74 (0.01) | 1.56 (<0.01) |
| **CSF p-tau181** | 68.85 (0.25) | 78.59 (0.27) | 70.88 (0.25) | 71.13 (0.25) | 0.02 (<0.01) | 2.11 (<0.01) | 1.37 (<0.01) |
| **CSF p-tau181/Aβ42** | 144.51 (0.41) | 145.84 (0.41) | 104.54 (0.37) | 96.76 (0.32) | 0.28 (<0.01) | 4.31 (0.02) | 2.61 (0.11) |

CSF: Cerebrospinal fluid; Aβ: beta-amyloid

**Supplementary Table 3 – SPM results for the main effects of CSF p-tau/Aβ42 on Gray Matter Volume**

| **t-value** | **C. Size [k]** | **p-value** | **pFWE** | **pFDR** | **x** | **y** | **Z** | **Brain area (AAL)** |
| --- | --- | --- | --- | --- | --- | --- | --- | --- |
| 4.516 | 712 | 0.0000051 | 0.132 | 0.297 | 17 | 14 | 18 | Caudate_R |
| 4.429 | 822 | 0.0000074 | 0.178 | 0.297 | 12 | -77 | 18 | Calcarine_R |
| 4.362 | 459 | 0.0000098 | 0.221 | 0.297 | 26 | -86 | 41 | Occipital_Sup_R |
| 4.238 | 412 | 0.0000164 | 0.322 | 0.331 | -17 | -3 | 26 | Caudate_L |
| 4.206 | 1917 | 0.0000187 | 0.351 | 0.331 | -47 | 0 | -2 | Temporal_Sup_L |
| 4.170 | 488 | 0.0000217 | 0.388 | 0.331 | -44 | -29 | -26 | Temporal_Inf_L |
| 4.079 | 107 | 0.0000313 | 0.488 | 0.401 | 18 | 68 | 15 | Frontal_Sup_R |
| 4.012 | 1221 | 0.0000409 | 0.566 | 0.413 | 63 | -57 | -3 | Temporal_Inf_R |
| 3.960 | 79 | 0.0000501 | 0.628 | 0.413 | 27 | 60 | 24 | Frontal_Mid_R |
| 3.932 | 163 | 0.0000560 | 0.662 | 0.413 | -20 | 42 | 29 | Frontal_Mid_L |
| 3.735 | 504 | 0.0001185 | 0.865 | 0.413 | -54 | -51 | 48 | Parietal_Inf_L |
| 3.716 | 239 | 0.0001274 | 0.881 | 0.413 | -48 | 11 | 33 | Precentral_L |
| 3.679 | 208 | 0.0001461 | 0.907 | 0.413 | -44 | -8 | -38 | Temporal_Inf_L |
| 3.599 | 358 | 0.0001957 | 0.950 | 0.438 | 42 | 14 | 0 | Insula_R |
| 3.498 | 60 | 0.0002824 | 0.982 | 0.492 | 3 | -56 | 66 | Precuneus_R |
| 3.381 | 644 | 0.0004252 | 0.996 | 0.567 | -44 | 18 | -6 | Frontal_Inf_Orb_L |
| 3.361 | 114 | 0.0004548 | 0.997 | 0.567 | 36 | 45 | 36 | Frontal_Mid_R |
| 3.310 | 91 | 0.0005427 | 0.999 | 0.603 | 66 | -12 | 29 | Postcentral_R |
| 3.308 | 85 | 0.0005455 | 0.999 | 0.603 | 0 | 62 | -14 | Rectus_L |
| 3.298 | 61 | 0.0005658 | 0.999 | 0.606 | 56 | 26 | 24 | Frontal_Inf_Tri_R |
| 3.294 | 122 | 0.0005731 | 0.999 | 0.606 | 45 | -57 | -23 | Temporal_Inf_R |
| 3.256 | 68 | 0.0006518 | 1.000 | 0.631 | 48 | -32 | 50 | Parietal_Inf_R |
| 3.241 | 91 | 0.0006848 | 1.000 | 0.643 | -50 | -23 | 30 | Postcentral_L |
| 3.227 | 120 | 0.0007177 | 1.000 | 0.656 | 0 | -62 | -6 | Vermis_4_5 |
| 3.210 | 205 | 0.0007583 | 1.000 | 0.675 | -23 | -63 | -6 | Lingual_L |
| 3.143 | 113 | 0.0009460 | 1.000 | 0.705 | 47 | -24 | -27 | Temporal_Inf_R |
| 3.098 | 171 | 0.0010971 | 1.000 | 0.718 | -18 | 54 | 15 | Frontal_Sup_L |
| 3.091 | 88 | 0.0011199 | 1.000 | 0.718 | -27 | -89 | 8 | Occipital_Mid_L |
| 3.076 | 77 | 0.0011757 | 1.000 | 0.718 | -44 | -59 | 56 | Parietal_Inf_L |
| 3.000 | 60 | 0.0015013 | 1.000 | 0.733 | 9 | -18 | 77 | Paracentral_Lobule_R |
| 2.940 | 131 | 0.0018070 | 1.000 | 0.770 | -35 | -39 | -26 | Cerebelum_6_L |
| 2.840 | 66 | 0.0024602 | 1.000 | 0.833 | 45 | 11 | -35 | Temporal_Pole_Mid_R |

**Supplementary Table 4 – SPM results for the interaction between CSF p-tau/Aβ42 and age status on Aβ deposition (i.e., Aβ PET)**

| **t-value** | **C. Size [k]** | **p-value** | **pFWE** | **pFDR** | **x** | **y** | **Z** | **Brain area (AAL)** |
| --- | --- | --- | --- | --- | --- | --- | --- | --- |
| 5.969 | 69240 | 0.0000000 | 0.000 | 0.003 | 56 | -32 | 12 | Temporal_Sup_R |
| 3.918 | 145 | 0.0000600 | 0.577 | 0.057 | 20 | -8 | 68 | Frontal_Sup_R |
| 3.825 | 83 | 0.0000859 | 0.686 | 0.074 | 38 | -22 | 46 | Postcentral_R |
| 3.749 | 76 | 0.0001140 | 0.768 | 0.085 | 32 | -8 | 54 | Precentral_R |
| 3.700 | 81 | 0.0001370 | 0.816 | 0.093 | 20 | -42 | 58 | Postcentral_R |

**Supplementary Table 5 – SPM results for the interaction between CSF p-tau/Aβ42 and sex status on Aβ deposition (i.e., Aβ PET)**

| **t-value** | **C. Size [k]** | **p-value** | **pFWE** | **pFDR** | **x** | **y** | **Z** | **Brain area (AAL)** |
| --- | --- | --- | --- | --- | --- | --- | --- | --- |
| 6.791 | 26885 | 0.0000000 | 0.000 | 0.000 | 50 | -78 | -14 | Occipital_Inf_R |
| 6.361 | 747 | 0.0000000 | 0.000 | 0.000 | -48 | -72 | -34 | Cerebelum_Crus1_L |
| 6.233 | 736 | 0.0000000 | 0.000 | 0.000 | 50 | -70 | -34 | Cerebelum_Crus1_R |
| 5.916 | 87 | 0.0000000 | 0.000 | 0.001 | 2 | -48 | -72 | Cerebelum_9_R |
| 5.301 | 608 | 0.0000001 | 0.004 | 0.004 | -38 | -64 | 36 | Angular_L |
| 4.410 | 262 | 0.0000082 | 0.144 | 0.034 | 18 | 18 | 18 | Caudate_R |
| 4.272 | 243 | 0.0000145 | 0.225 | 0.045 | -16 | 20 | 14 | Caudate_L |
| 3.882 | 86 | 0.0000688 | 0.609 | 0.112 | 22 | -14 | 54 | Precentral_R |
| 3.860 | 89 | 0.0000751 | 0.636 | 0.116 | -2 | -30 | -48 | Cerebelum_9_L |
| 3.730 | 184 | 0.0001225 | 0.779 | 0.137 | -34 | 2 | 42 | Precentral_L |
| 3.498 | 62 | 0.0002844 | 0.949 | 0.230 | -36 | -80 | 30 | Occipital_Mid_L |
| 3.330 | 89 | 0.0005114 | 0.991 | 0.312 | -10 | 52 | 14 | Frontal_Sup_Medial_L |
| 3.243 | 137 | 0.0006847 | 0.997 | 0.359 | 36 | -70 | 16 | Occipital_Mid_R |
| 3.029 | 62 | 0.0013740 | 1.000 | 0.503 | 62 | 4 | -10 | Temporal_Sup_R |

**Supplementary Table 6 – SPM results for the interaction between CSF p-tau/Aβ42 and *APOE*-ε4 status on Aβ deposition (i.e., Aβ PET)**

| **t-value** | **C. Size [k]** | **p-value** | **pFWE** | **pFDR** | **x** | **y** | **Z** | **Brain area (AAL)** |
| --- | --- | --- | --- | --- | --- | --- | --- | --- |
| 3.621 | 90 | 0.0001833 | 0.875 | 0.970 | -30 | -14 | -24 | Parahippocampal_L |
| 3.286 | 130 | 0.0005940 | 0.995 | 0.970 | 24 | -56 | 54 | Parietal_Sup_R |
| 3.071 | 91 | 0.0012053 | 1.000 | 0.970 | 44 | -44 | 36 | SupraMarginal_R |

**Supplementary Table 7 – SPM results for the interaction between CSF p-tau/Aβ42 and age on Gray Matter Volume**

| **t-value** | **C. Size [k]** | **p-value** | **pFWE** | **pFDR** | **x** | **y** | **Z** | **Brain area (AAL)** |
| --- | --- | --- | --- | --- | --- | --- | --- | --- |
| 6.144 | 4241 | 0.0000000 | 0.000 | 0.003 | 33 | -59 | -41 | Cerebelum_Crus1_R |
| 6.019 | 9150 | 0.0000000 | 0.000 | 0.003 | 48 | 0 | -29 | Temporal_Mid_R |
| 5.312 | 2138 | 0.0000001 | 0.006 | 0.016 | -45 | -8 | -35 | Temporal_Inf_L |
| 5.249 | 1298 | 0.0000002 | 0.007 | 0.016 | 39 | -26 | 39 | Postcentral_R |
| 5.120 | 3631 | 0.0000003 | 0.013 | 0.020 | -24 | -69 | -30 | Cerebelum_Crus1_L |
| 5.115 | 3150 | 0.0000003 | 0.013 | 0.020 | -48 | 8 | -6 | Temporal_Sup_L |
| 4.916 | 517 | 0.0000008 | 0.029 | 0.035 | 15 | -89 | 24 | Occipital_Sup_R |
| 4.798 | 1131 | 0.0000015 | 0.047 | 0.046 | 20 | -5 | -12 | Hippocampus_R |
| 4.712 | 957 | 0.0000021 | 0.065 | 0.056 | -6 | -69 | 23 | Calcarine_L |
| 4.658 | 500 | 0.0000027 | 0.079 | 0.061 | -24 | -59 | -6 | Lingual_L |
| 4.592 | 719 | 0.0000036 | 0.101 | 0.067 | -23 | 36 | 29 | Frontal_Sup_L |
| 4.400 | 710 | 0.0000083 | 0.195 | 0.106 | -3 | 33 | -15 | Rectus_L |
| 4.213 | 500 | 0.0000182 | 0.345 | 0.178 | 9 | -12 | 45 | Cingulum_Mid_R |
| 4.150 | 979 | 0.0000235 | 0.408 | 0.196 | -51 | -30 | 44 | Parietal_Inf_L |

**Supplementary Table 8 – SPM results for the interaction between CSF p-tau/Aβ42 and Aβ status on Gray Matter Volume**

| **t-value** | **C. Size [k]** | **p-value** | **pFWE** | **pFDR** | **x** | **y** | **Z** | **Brain area (AAL)** |
| --- | --- | --- | --- | --- | --- | --- | --- | --- |
| 4.007 | 320 | 0.0000417 | 0.572 | 0.991 | -41 | 12 | -12 | Insula_L |
| 3.946 | 256 | 0.0000531 | 0.645 | 0.991 | -51 | 23 | -2 | Frontal_Inf_Orb_L |
| 3.938 | 676 | 0.0000546 | 0.654 | 0.991 | 41 | 11 | -14 | Insula_R |
| 3.610 | 63 | 0.0001882 | 0.945 | 0.991 | -9 | -62 | 35 | Precuneus_L |
| 3.608 | 127 | 0.0001899 | 0.946 | 0.991 | -6 | -89 | 11 | Calcarine_L |
| 3.568 | 80 | 0.0002194 | 0.963 | 0.991 | -12 | 44 | 0 | Cingulum_Ant_L |
| 3.430 | 97 | 0.0003591 | 0.992 | 0.991 | -24 | 21 | 60 | Frontal_Mid_L |
| 3.314 | 58 | 0.0005363 | 0.999 | 0.991 | -38 | -6 | -18 | Hippocampus_L |
| 3.280 | 152 | 0.0006017 | 0.999 | 0.991 | -33 | -89 | 9 | Occipital_Mid_L |
| 3.155 | 115 | 0.0009100 | 1.000 | 0.991 | 53 | 44 | -9 | Frontal_Inf_Orb_R |
| 2.915 | 102 | 0.0019541 | 1.000 | 0.991 | 15 | 42 | 17 | Cingulum_Ant_R |
| 2.897 | 53 | 0.0020665 | 1.000 | 0.991 | -59 | 6 | -17 | Temporal_Mid_L |

**
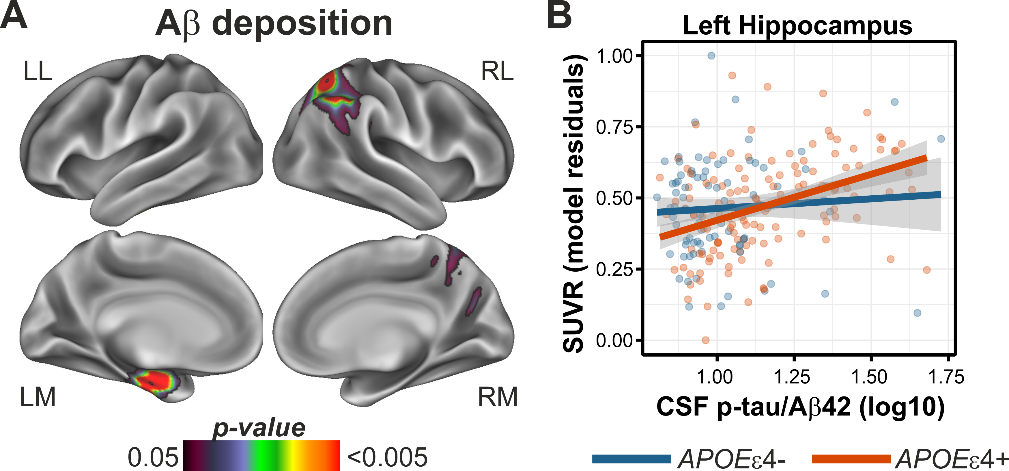
**

**Supplementary Figure 1 – *APOE*-ε4 modified the association between CSF p-tau/Aβ42 and cortical Aβ deposition.** For the same level of CSF p-tau/Aβ42, *APOE*-ε4 carriers compared to non-carriers, displayed a greater Aβ deposition in the left anterior hippocampus. **A)** Surface rendering of the significant cluster where an interaction between CSF p-tau/Aβ42 was found. Statistical analyses were performed using linear regression, and the projected p-values correspond their respective F-test. **B)** Group scatterplot showing the significant interaction between CSF p-tau/Ab42 and *APOE*-ε4 binary status. Each data point represents an individual subject (N=221). Statistical analyses were performed using linear regression (p=<0.05)

**SUPPLEMENTARY REFERENCES**

Buschke, H., Mowrey, W.B., Ramratan, W.S., Zimmerman, M.E., Loewenstein, D.A., Katz, M.J., Lipton, R.B., 2017. Memory Binding Test Distinguishes Amnestic Mild Cognitive Impairment and Dementia from Cognitively Normal Elderly. Arch Clin Neuropsychol 32, 1037-1038.

Mowrey, W.B., Lipton, R.B., Katz, M.J., Ramratan, W.S., Loewenstein, D.A., Zimmerman, M.E., Buschke, H., 2017. Memory Binding Test Predicts Incident Amnestic Mild Cognitive Impairment. J Alzheimers Dis 58, 951-952.

Papp, K.V., Amariglio, R.E., Mormino, E.C., Hedden, T., Dekhytar, M., Johnson, K.A., Sperling, R.A., Rentz, D.M., 2015. Free and cued memory in relation to biomarker-defined abnormalities in clinically normal older adults and those at risk for Alzheimer's disease. Neuropsychologia 73, 169-175.

Wechsler, D., 2012. WAIS-IV, Escala de inteligencia de Wechsler para adultos-IV. Pearson, Madrid
